# Hybrid Ontology for Semantic Information Retrieval Model Using Keyword Matching Indexing System

**DOI:** 10.1155/2015/414910

**Published:** 2015-04-01

**Authors:** K. R. Uthayan, G. S. Anandha Mala

**Affiliations:** ^1^Department of Information Technology, SSN College of Engineering, Chennai 603110, India; ^2^Department of Computer Science Engineering, Easwari Engineering College, Chennai 600089, India

## Abstract

Ontology is the process of growth and elucidation of concepts of an information domain being common for a group of users. Establishing ontology into information retrieval is a normal method to develop searching effects of relevant information users require. Keywords matching process with historical or information domain is significant in recent calculations for assisting the best match for specific input queries. This research presents a better querying mechanism for information retrieval which integrates the ontology queries with keyword search. The ontology-based query is changed into a primary order to predicate logic uncertainty which is used for routing the query to the appropriate servers. Matching algorithms characterize warm area of researches in computer science and artificial intelligence. In text matching, it is more dependable to study semantics model and query for conditions of semantic matching. This research develops the semantic matching results between input queries and information in ontology field. The contributed algorithm is a hybrid method that is based on matching extracted instances from the queries and information field. The queries and information domain is focused on semantic matching, to discover the best match and to progress the executive process. In conclusion, the hybrid ontology in semantic web is sufficient to retrieve the documents when compared to standard ontology.

## 1. Introduction

The difficulty of information storage space and retrieval has concerned escalating special treatment since 1940. The difficulty affirms that huge quantities of information to be stored and the relevant information should be precise. An enormous contract of research work has been completed to offer speedy and intellectual retrieval methods. To the research concern of digital libraries, several indeed contain information storage and retrieval troubles, such as logging and textual penetrating. Conversely, the difficulty of successful repossession continues mostly vague. Civilizing the usefulness is a significant ambition for the research of information retrieval system. Identifying the concepts or effort of the user is the major complicated obsession for relevant documents searching from a huge amount of information. For the user using common terms of queries for searching, an information retrieval system will not provide functional and detailed answers. The domain information of documents and cognition of the user are thus major for the retrieval of relevant documents information.

The research on combining the methods of ontology and information retrieval for semantic web is emerging in recent times. To explore the relevant information for the users need, a conventional method is introduced by entrenching ontology into information retrieval. If the investigated information is enclosed beneath the knowledge domain of user's concepts, the motivation increases the probability of relevance. Therefore, the efficiency possibly enhanced. The challenges of implanting domain knowledge into information retrieval system are as follows.

1. What is the apposite information retrieval model?
2. How to execute and build ontology?
3. How to discover the relevant documents by ontology?

The semantic web is build for current web extension where the information has well defined meaning and enabling cooperation between people and computers. Because of this well-defined structure, humans and even machines will work in coperation. The standard fuzzy ontology is a technique which is used in information retrieval where the calculation of relationship among the concepts are done using membership values. From domain's uncertainty data, generation of fuzzy ontology automatically is highly desirable. This research explores hybrid fuzzy ontology-based information retrieval models in semantic web and gossip about the achievement and authority of applying proposed ontology containing common field knowledge and fuzzy concepts fabricated from the stored documents automatically. For mapping, the generated fuzzy ontology to semantic representation Web Ontology Language (OWL) is used.

This research work is organized as follows: The related work is reviewed in Section 2. The proposed ontology-based information retrieval model is depicted in Section 3. The experiments and discussion on the results are described in Section 4. Finally, conclusion is given in Section 5.

## 2. Related Materials

Tho et al. [1] proposed the FOGA (Fuzzy Ontolology Generation frAmework) in which fuzzy ontology is generated on vague information automatically. A fuzzy-based method is described for integrating database attributes to the ontology. They converse about approximating reasoning for additional enhancement of the ontology. de Maio et al. [2] described an approach by analyzing the web resource collection for automatic fuzzy ontology elicitation. This approach applicability is validated by web domain case study. Abulaish et al. [3] recommended a fuzzy ontology generation framework in which instead of concept descriptor, the possession quantity is encoded using fuzzy membership function. The Fuzzy Formal Concept Analysis (FFCA) which is generalization of Formal Concept Analysis (FCA) used for sculpting vagueness information. Formica [4] showed the FFCA amalgamation with rough set theory to complete semantic web exploration and detection of information in the web. Chahal et al. [5] presented a similarity comparison scheme of semantic web document which relies instances between keywords in documents and also the relationship in the web pages which exists between concepts amalgamation.

Formica [6] proposed a similarity measure for FFCA. This FFCA is usually intended for restricted audience and addressed at technical level, although, it becomes very interesting for semantic web development by supporting different activities. The development of ontologies manually is a time consuming and cumbersome task. Zhang et al. [7] planned an approach and an automated tool from Fuzzy Object Oriented Database (FOOD) models for constructing the fuzzy ontologies. This ontology plays an important role for the development of new strategies of knowledge based systems and in supporting the automated process for accessing information. So, de Maio et al. [8] presented an ontology-based retrieval approach, which supports data organization and visualization and provides a friendly navigation model.

To design information retrieval system, the major challenges for researchers and developers is the method of sharing and searching the information with emergence of web. Kohli and Gupta [9] surveyed the challenges in information retrieval and solve those challenges with the help of fuzzy concept. Aloui et al. [10] have presented a semiautomatic method for fuzzy ontology extraction and design (FOD). The method is based on conceptual clustering, fuzzy logic, and formal concept analysis (FCA). The core of ontology is represented as a set of fuzzy rules. To validate the proposed approach, they used Protégé 4.3 that supports the fuzzy concept and automatically generate the script in OWL-2 language.

Sometimes irrelevant information is retrieved on the semantic web but it is meaningful, and with ontology mapping, the relevance can be improved. Kandpal et al. [11] described a new technique for ontology mapping. Two various ontologies of a domain are considered, and the concepts which are similar to each other are retrieved, that is, ontology alignment. The similarity is calculated if the concepts are not matched even when term is expanded. One of the challenges in information retrieval is providing accurate answers to a user's question often expressed as uncertainty words. Rani et al. [12] presented a hybrid approach for a semantic question answering retrieval system using ontology similarity and fuzzy logic to retrieve collection of documents. Fuzzy scale uses fuzzy type 1 for documents and fuzzy type 2 for words to prioritize answers.

Recently, the data originated from multiple types of sources includes the mobile devices, individual archives, sensors, social networks, enterprises, and cameras; Internet of things, software logs, and health data have led to one of the most challenging research concerns of the big data era. So, Xu et al. [13] suggested the basic blocks of the Knowles system, resources representation, semantic relations mining, and semantic linking news events, and it does not need data contributors to pursue semantic standards such as RDF or OWL, which is a semantics loaded self-categorized network.

Liu et al. [14] proposed a technique in effective manner for organizing the associated multimedia resources and for semantic link network model which is used for organizing multimedia network. The community cloud computing is a promising and emerging model for a particular community with general concerns, such as compliance, security, and jurisdiction. Selecting the best group of community clouds that are the most economy and communication effective and trusted to complete a difficult task is extremely challenging. To deal with this problem Hao et al. [15] formulate computational model multi-community-cloud collaboration, namely, MG3. The proposed model is then optimized from four aspects: minimize the sum of monetary and access cost, make the most of security level agreement and trust among the community clouds.

So, the study of related works motivates the semantic matching technique by combining the fuzzy ontology with keyword matching to retrieve the relevant information.

## 3. Research Methodology

The hybrid ontology approach to query interpretation is on the aspiration of generating more than one specific planned query from a given keyword. This research refers to every produced query as an elucidation. The proposed model uses a hybrid fuzzy ontology for semantic relevant document retrieval. It semantically repossesses a position of related documents along with users query esteeming the emphasized sector or domain. It can be used to retrieve every category of documents in a particular domain written in all languages. The proposed information retrieval models and their major components are a set of annotated documents, user's queries, retrieval engine, and ranking module. The relationships between concepts are built using ontology terms and NLP techniques. The relationships and natural-language synonyms represents the entities which completes the ontology by considering the key technique of NLP.

**Figure 1:**
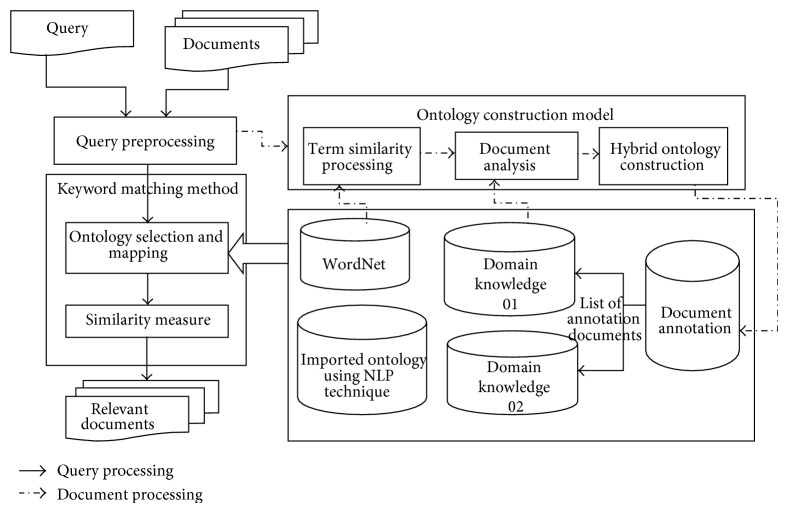
Hybrid ontology for information retrieval.

As demonstrated in Figure 1, the proposed hybrid ontology-based information retrieval model encloses the following modules.

*(i) Query Preprocessing.* Query preprocessing is a necessary step for extracting terms and aspects. The important function of this section is to eliminate the insignificant words and filter the major keywords. 

*(ii) Ontology Construction Methods.* This module tries to build fuzzy taxonomy on behalf of ontology from documents without human intervention. In order to produce ontology professionally, the development process is separated into three steps: term similarity processing, document analysis, and clustering algorithm. 

*(iii) Matching Method. *It is the major retrieval mechanism. The related documents usually recovered and ranked using similarity matching. 

*(iv) Ontology Base. *Various forms of ontology are adopted in the anticipated model such as WordNet; users' field information constructed manually and automatically creates the fuzzy taxonomy. 

*(v) Ranking the Resulted Documents. *The escalating weight is intended for every permutation of words derived from enhanced matching algorithm. The most excellent document obtains the least score. The documents are assembled in mounting order according to their collective score. The ranked listing of appropriate documents is then demonstrated to the user in the matching order. 

*(vi) Document Annotation for Retrieving Information*. From the domain knowledge the documents are annotated with concept by creating annotation class. By using domain expert the annotations are be created automatically. Each case is differentiated using the manual subclass or with automatic annotations. A valid outcome of document for an exactitude oriented keyword query is observed using two events: (i) the designed search assignment of the user, presenting the query and (ii) the semantic documents are satisfied. To reach the relevant document semantics, this research proposes an alternative to information extraction techniques for recognizing states of entities and relationships in a text document. Every declaration is known as annotation and a formatted data accumulate including the intact of the extorted annotations is called an annotation store.

Usually, for information retrieval system the documents are processed in two phases: document processing and query processing. In document processing stage, by using textual preprocessing the documents are processed to gain imperative stipulations and features for representing the documents. The conditions then are applied to construct fuzzy taxonomies from side to side of the ontology building techniques. The concepts contain definitions and instances which is given by the textual description of WordNet. WordNet can be satisfied as a moderately structured synonym store.

There are three databases in WordNet, noun is the initial one, verbs is second database, adjectives and adverbs are the final one. “Synsets” is a set of synonyms which designate a concept or a sagacity of a set of terms. Synsets available make diverse semantic relations for instance synonymy (similar) and antonymy (opposite), hypernymy (super concept)/hyponymy (subconcept) (also known as a hierarchy/taxonomy), meronymy (part-of), and holonymy (has-a). Depending on the grammatical category, the semantic relatives with the synsets will vary. The following sections discuss about document processing and information retrieval using standard fuzzy ontology framework.

### 3.1. Fuzzy Ontology Framework for Information Retrieval

**Figure 2:**
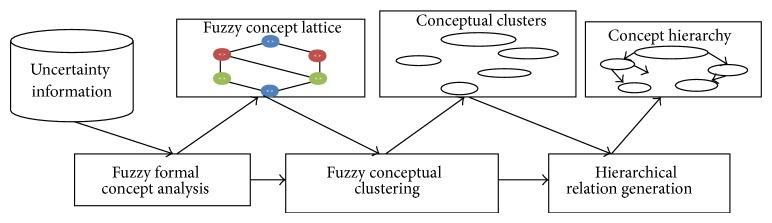
The traditional FOGA framework.

In FOGA [1] construction, a fuzzy logic offers a hypothetical framework for the demonstration and management of the information with their deficiencies. It does not undertake to remove them; on the contrary, it aims to protect them. Its target is consequently to construct settings of demonstration and behavior of knowledge efficiently, and it is stimulated from the human intellectual process. It slopes on the mathematical fuzzy sets theory. This presumption is a growth of the common set theory for investment groups described in a vague approach. The traditional FOGA consists of the following components (see Figure 2).

*(a) Fuzzy Formal Concept Analysis.* From a database restraining unsecured data, it assembles fuzzy context. Additionally, it will also execute* fuzzy formal concepts *from the* fuzzy formal context *and categorizes the created concepts as a* fuzzy concept lattice*. 

*(b) Fuzzy Conceptual Clustering.* It groups concepts on the* fuzzy concept lattice *and executes* conceptual clusters*. The clustering method is evaluated from fuzzy information and integrated into the web using fuzzy logic. 

*(c) Hierarchical Relation Generation.* It produces hierarchical relationship between conceptual clusters to build a* concept hierarchy*.

In Algorithm 1, based on the hypothesis the conceptual clusters are derived that if a formal concept *B* is similar to *A*, then conceptual cluster *R* will be based on formal concept *A* and its sub concept *B*. The similarity between two concepts is determined by similarity confidence threshold *T*_*s*_.

To characterize vague information, the restriction of fuzzy logic will be integrated into ontology. Characteristically, fuzzy ontology is constructed from a predetermined concept hierarchy. On the other hand, a complicated and tedious process is assembling the concept hierarchy for a particular domain. To overcome this difficulty, the FOGA is implemented for generating fuzzy ontology automatically on information uncertainty.

### 3.2. Keyword Matching Ontologies

Ducatel et al. [16] broadly described ontology-based queries for query generation and matching of service representatives. However, services may also desire to illustrate themselves with free text, such as with keywords that are not already specified in the ontology. In order to be capable of handling, it requires a selected way for the ontology to handle keywords and concepts. Collaborative information is a method of exploiting this data for the benefit of other users, where frequent queries (from different users) are associated with valuable outcomes.

The equal relationships with keywords is segregated for illustrating the semantic conceptions among documents in term comparison processing. The quantity of identical association can be calculated by semantic comparison calculating process. In a set of procedures, based on WordNet the word comparison between keywords is intended through similarity measure.

Initially in document investigation, the important keywords are selected from the documents as the specific keyword space *K*. Then, the chosen keywords are allocated into many attributes of the keyword space *K*. Let *A* be the set of attributes in *K*, *A*_*m*_ = {*k*_*m*,1_, *k*_*m*,2_,…, *k*_*m*_, *p*_*m*_}, where *A*_*m*_ ∈ *A* and *k*_*m*,*n*_ ∈ *K*.  Algorithm 2 presents the demonstration in keyword space *K* which is measured through a subsequent algorithm for collections of document *D*.

**Figure 3:**
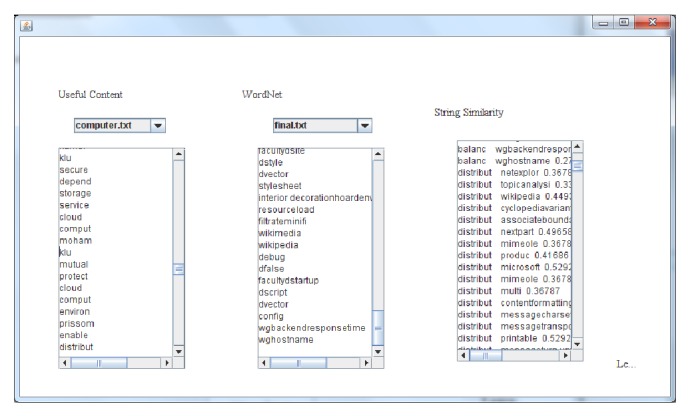
The similarity strings.

As the keywords in* keyword space* are measured, the hybrid fuzzy matching technique is well designed to assemble fuzzy classification for every set of attributes. The created fuzzy arrangement matching to the domain of particular attributes is then accepted as the ontology applied to retrieve relevant information. The matching strings are shown in Figure 3.

According to the professional field (computer science), the ontology model is constructed which is depicted in Algorithm 3.

### 3.3. Combining Hybrid Fuzzy Ontology Generation Framework and Keyword Matching Ontologies

After splitting the query into meaningful words, each word should be checked against the ontology. The entire amalgamation of words is in use for processing. Scrupulous domain ontology is received to verify whether the declaration is to provide ontology. If persuaded, then the association of the words is obtained into the deliberation. The points are described for matching ontologies and the rules used to group related concepts together are listed below (parents-superset and child-subset).

- The parent conception demonstrates the perspective of the concept, from this parent each matching concept are collected.
- Matching concepts with similar parent are controlled by individual score, ought to be located jointly under individual score.
- Each series of parent-child associated matching concepts that demonstrates the context of the series must end in a non-matching concept.
- Unconnected groups are attached together as afforest, prepared by the highest score of the group.
- If the parents have the children with similarity then they will acquire the privileged of two portions and are connected together.

In consequence the amalgamation of mutual hybrid FOGA and keyword matching with an elucidation of a keyword query is set together by individually matching the query terms in the keyword query against the elements of annotation store. An annotation store *S* = (*T*, *O*, *D*) consists of a position of types *T* (signify doc, docx, pdf, etc.), a set of objects *O*, and exceptional distinguished sort *D* ∈ *T* such that, for every *x* ∈ *O*, type(*x*) ∈ *T*. Further, for every object *x* ∈ *O*, also type(*x*) = *D*; otherwise there survives an element doc with type(*x*.doc) = *D*. Given an annotation store *S* = (*T*, *O*, *D*) and a query term *K* where *S* is the type of document and each added type is an annotation type in *S*. In the above, object *x* is represented by type(*x*). A document attribute is enclosed for each attribute which look up the document from where the objects are extracted. This annotation store of the path can be of any expression of *T*.*a*_1_ ⋯ *a*_*m*_, where legitimate attribute of type *T* is represented as *a*_1_, type attribute (*T*.*a*_1_) is *a*_2_ and so on.

This research work envisages the following three forms of matches.

*(i) Type Match*. If the particular or selection name of its significance is matched by *k*, then *k* matches a type *T* ∈ *T*. For example, the keywords “phone”, “contact” and “number” may all match the type Phone Number, if all three keywords have been defined as synonyms of this concept. In common, this research assumes that the input to the precision oriented retrieval system is the set of synonyms which is associated with each type.

*(ii) Path Match*. Matches not in favor of paths are calculated in an analogous approach using the matching set of synonyms. The path match contains maxQDist-vector and scalar parameter, Δ*q*-query, and Δ*r*-search collection.

Algorithm 4 uses this constraint to avoid big nonmatching gaps between consecutive matching points. This algorithm considers the maxQDist as the maximum elapsed time in either time series. Moreover, given that the query is processed sequentially in time (i.e., *tq*_*i*_ < *tq*_*i*+1_  ∀*i*), paths that do not comply with this constraint are removed from Δ*T* (function “process&extract()”), as it is ensured that they will no longer comply with the constraint. The removed paths are then evaluated in terms of minimum length, number of matching points, and score to determine if they can be considered a good match between both time series (*tq*_*i*_, *tr*_*j*_).

For instance, as the synonym “fone” is connected with the concept PhoneNumber, then TypePath index maps “fone” to the type PhoneNumber, to the path Author-Phone.phone, and so on. As such, the synonyms “callin,” “dial-in,” “concall,” and “conferencecall” are mapped to the type ConferenceCall. The keyword “tom” has a value match with Author.name, AuthorPhone.author.name indicating that “tom” has appeared as the name of the author of an email, as the name of a person who was declared in the signature block of an email, and so forth.

*(iii) Value Match.* To conclude, matches not in favor of minute values are calculated with contrasting *k* next to the rest of minute values connected with every path in the annotation store. The value matching makes use of domain checks to calculate the relationship computed among phrases. At any time constraint value-sets are present, we can enhance our knowledge of the domain, as such constraints turn to be precious when evaluating two terms that do not precisely match through their labels.

The next step of comparison measure retrieves and ranks the relevant documents from the document database. In the beginning, the ontology of query preferred form the initial step (in Algorithm 5), is used to regulate the weights of documents. The method of computing adjusted weights for *H*_*q*_ is demonstrated as follows:

(1) $$\begin{array}{c} {\overset{⃑}{d}}_{i}^{\prime }=\underset{k_{d}\in d_{i}}{\sum }\max\left(\underset{k_{q}\in H_{q}}{\sum }R\left(k_{q},k_{d}\right)\right)\times w_{i,d}, \end{array}$$

where *w*_*id*_ is the weight of document and *d*_*i*_ presented in term *k*_*d*_. *k*_*q*_ is the terms of *H*_*q*_. Finally, the comparison measure is computed with the following function:

(2) $$\begin{array}{c} \text{Sim}\left({\overset{⃑}{d}}_{i}^{\prime },\overset{⃑}{q}\right)=\max\left(\underset{s_{r}\in S}{\sum }\cos\left({\overset{⃑}{d}}_{i}^{\prime },\overset{⃑}{q}\right)\times s_{r}\right), \end{array}$$

where *s*_*r*_ is the weights of nominated ontology. $\cos\left({\overset{⃑}{d}}_{i}^{\prime },\overset{⃑}{q}\right)$ is the cosine comparison. For instance, a query “Fishing ferry in South Africa” can be symbolized as {“fish”, “ferry”, “in”, “South Africa”}. The term “south africa” is mapped into the concept “s africa” of the ontology *H* “Location” and *s* “Location” = 1. The ontology of query *H*_*q*_ is mapped.

## 4. Experimental Results

This section described the experimental setup for hybrid FOGA using keyword matching to retrieve the relevant information and ranking the documents automatically. The dataset is constructed using list of abstracts selected from 1000 documents which are all collected from the web. Initially the documents are updated to the FOGA framework with preprocessed information. The elimination of stop words and operations of stemming are performed. The weight estimation process is done with term analysis and semantic analysis tasks. The related journals are collected for the fuzzy ontology from the web. Using HTML, the abstract pages are intended for manuscripts. The text document conversion is done by removing the HTML tag elements from the web documents and document information is maintained in separate files. The two most common and important metrics for information retrieval efficiencies are precision and recall. In consequence, this research work used these measures for the ontology presentation for evaluation. Precision and recall are described in terms of a set of retrieved documents (e.g., the list of documents listed through a web search engine for an uncertainty) and a group of relevant documents (e.g., the list of every document on the net that is applicable for a convinced area):

(3) Precision=#relevant  items  retrieved#retrieved  items=Prelevant ∣ retrieved,Recall=#relevant  items  retrieved#relevant  items=Pretrieved ∣ relevant,F-Measure=2·precision·recallprecision+recall.

The standard precision combines each query at recall level diagonally and calculates whole system performance approximately on a document/query capability.

For the sake of precision and recall, some researchers improve the architecture of inverted files. The authors move query keywords to semantic terms. But index tables still used keyword-based ones. To make the match easier, a new index table with semantic terms is proposed in this work.

The combination of standard ontology with FOGA techniques in this research, prescribes the solution for information retrieval using keyword matching indexing techniques. The *F*-measure indicates that the overall average performances of all relationships are similar, with a slight trend of higher *F*-measure for hybrid FOGA implementations.

**Figure 4:**
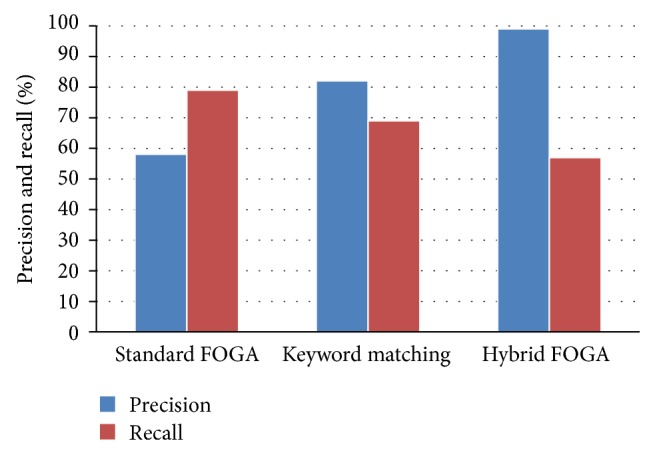
Showing the precision and recall for proposed hybrid FOGA.

**Figure 5:**
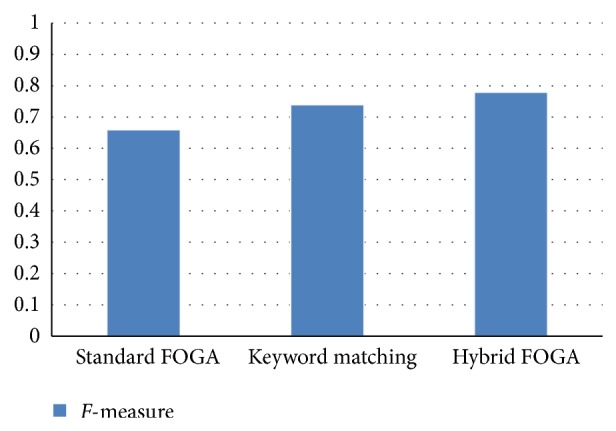
The *F*-measure for proposed hybrid FOGA.

Both Figures 4 and 5 represent the precision, recall and *F*-measure for information retrieval by comparing three schemes for fuzzy ontology framework. The hybrid technique has shown the best precision, recall and *F*-measure values in the FOGA framework. Our approach improve the classical methodology approach and the best documents are in the top of retrieved document list.

**Figure 6:**
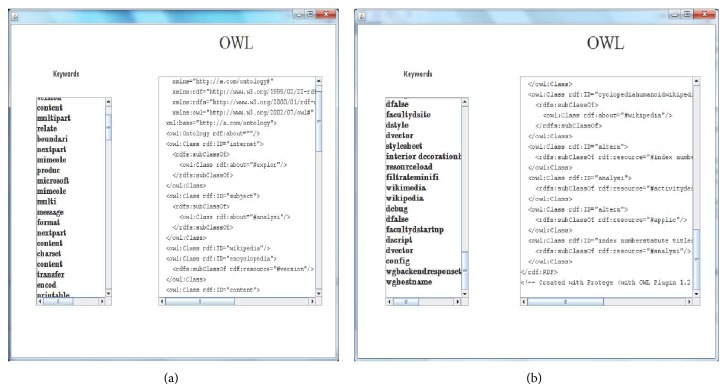
The hybrid fuzzy ontology.

To evaluate the proposed hybrid FOGA framework this research collected a set of 1,000 scientific documents in the research area “information retrieval.” There are two shortest goals general to all IR methods: (a) effectiveness: IR must be accurate (achieves what the user expects to observe in the answer); (b) efficiency: IR should be speedy (quicker than chronological scanning). The main goal of information retrieval is to possess relevant documents in response to user needs. The performance of ontology is evaluated with the research area hierarchy created using hybrid FOGA. Initially precision, recall and *F*-measure are calculated for information retrieval. If these parameters acquires the goodness, then the conceptual information are generated accurately. Thus, the performance of hybrid fuzzy ontologies is shown in Figure 6.

## 5. Conclusion

In this research, a latest approach for retrieving information successfully through implementation of hybrid ontology is discussed. This research presents a development in the hybrid ontology semantic information retrieval through (a) getting back a group of relevant documents semantic method using the proposed hybrid ontology, (b) dealing with the variety of field topics problem using hybrid concept view fuzzy ontology, and (c) ranking the end result set of documents according to *F*-measures which are relevance quantity with respect to uses query, confidence, and updating degree. So, this research proposed a hybrid ontology which integrates and takes advantages of SW and IR technologies to provide better search capabilities achieving a qualitative improvement by using keyword-based information retrieval. The future work in this part is possible to construct a document annotation algorithm using the proposed hybrid ontology. Furthermore, the hope of this research work motivates implementing fuzzy theory and neural network methods to build fuzzy ontology from unstructured data automatically.

## Figures and Tables

**Algorithm 1:**
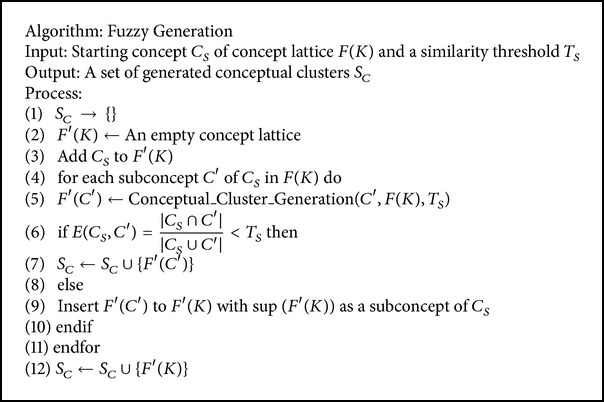
Fuzzy generation model.

**Algorithm 2:**
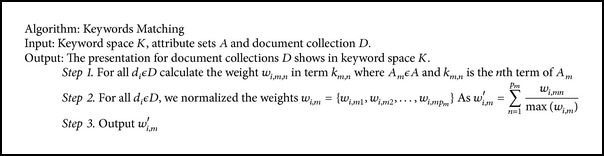
Keyword matching.

**Algorithm 3:**
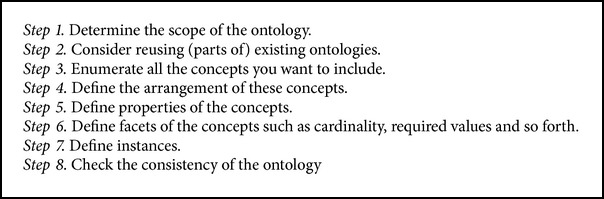
Steps for construction model.

**Algorithm 4:**
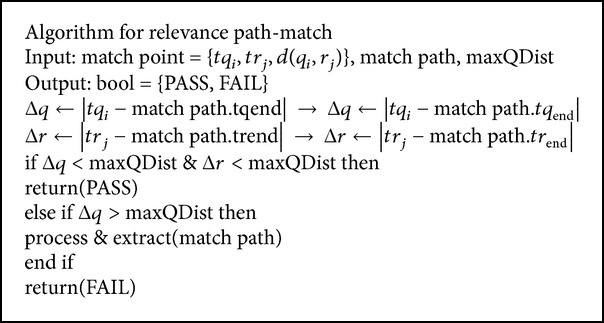
Relevance path match.

**Algorithm 5:**
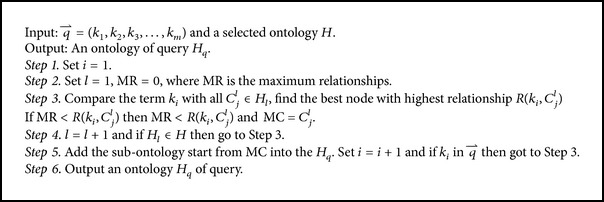
Hybrid ontology mapping.
